# Long-Lasting Effects of Oxy- and Sulfoanalogues of L-Arginine on Enzyme Actions

**DOI:** 10.1155/2013/407616

**Published:** 2013-10-24

**Authors:** Tatyana A. Dzimbova, Peter B. Milanov, Tamara I. Pajpanova

**Affiliations:** ^1^Institute of Molecular Biology, Bulgarian Academy of Sciences, 1113 Sofia, Bulgaria; ^2^Faculty of Mathematics and Natural Sciences, South-West University “Neofit Rilski”, 2700 Blagoevgrad, Bulgaria; ^3^Institute of Mathematics and Informatics, Bulgarian Academy of Sciences, 1113 Sofia, Bulgaria

## Abstract

Arginine residues are very important for the structure of proteins and their action. Arginine is essential for many natural processes because it has unique ionizable group under physiological conditions. Numerous mimetics of arginine were synthesized and their biological effects were evaluated, but the mechanisms of actions are still unknown. The aim of this study is to see if oxy- and sulfoanalogues of arginine can be recognized by human arginyl-tRNA synthetase (HArgS)—an enzyme responsible for coupling of L-arginine with its cognate tRNA in a two-step catalytic reaction. We make use of modeling and docking studies of adenylate kinase (ADK) to reveal the effects produced by the incorporation of the arginine mimetics on the structure of ADK and its action. Three analogues of arginine, L-canavanine (Cav), L-norcanavanine (NCav), and L-sulfoarginine (sArg), can be recognized as substrates of HArgS when incorporated in different peptide and protein sequences instead of L-arginine. Mutation in the enzyme active center by arginine mimetics leads to conformational changes, which produce a decrease the rate of the enzyme catalyzed reaction and even a loss of enzymatic action. All these observations could explain the long-lasting nature of the effects of the arginine analogues.

## 1. Introduction

Peptidomimetics have found wide application as bioavailable, biostable, and potent mimetics of naturally occurring biologically active peptides. l-Arginine has guanidinium group, which is positively charged at neutral pH and is involved in many important physiological and pathophysiological processes [1]. It has a very ionizable amino acid, and it is found most frequently buried in the protein interior [2–5]. Arginine residues are essential in a variety of biological processes, such as the regulation of conformation or redox potentials [6, 7]; viral capsid assembly [8]; electrostatic steering [9]; voltage sensing across lipid bilayers [10–12]; H+ transport [6, 13–15] and peptide translocation across bilayers [16, 17]. They also play a critical role at protein-protein interfaces [5, 16], in enzymatic active sites [3, 5, 18], and in variety of transport channels [19, 20]. More recent findings show that arginine-specific methylation of histones may cooperate with other types of posttransitional histone modification to regulate chromatin structure and gene transcription [21]. Proteins that methylate histones on arginine residues can collaborate with other coactivators such as nuclear receptors.

Enzymes are probably the most studied biological molecules. They constitute nature's toolkit for making and breaking down the molecules required by cells in the course of growth, repair, maintenance, and death. Virtually, every biological process requires an enzyme at some point. Enzymes are capable of carrying out complex transformations in aqueous solution, at biological temperatures and pH, and in a stereospecific and regiospecific manner, a feat seldom achieved by the best of organic chemists [3, 22]. Crystallographic and NMR studies of enzymes have shed light on the relationship between an enzyme's three-dimensional (3D) structure and the chemical reaction it performs. There are many complications, however, in assigning the functions of catalytic residues, due to the multistep nature of a chemical reaction. One residue can play more than one role and can be involved in different steps of the reaction.

**Figure 1:**
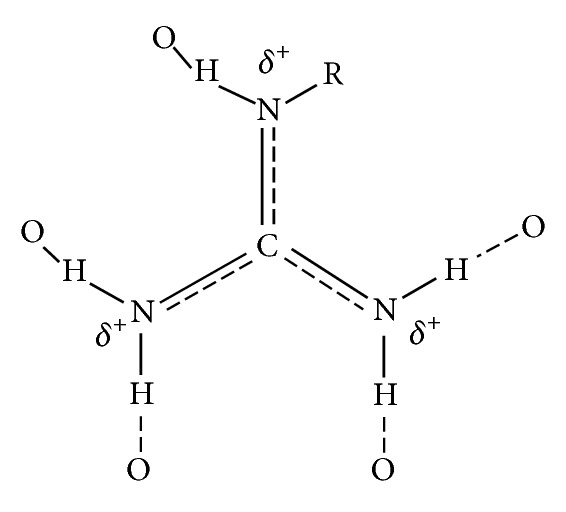
Schematic presentation of the hydrogen bond formation of the guanidinium group with 5 different oxygen atoms.

Arginine (Arg) is one of the most important residues in catalytic centers of many enzymes. Arg is in the 3rd place of the enzymatic frequency distribution and constitutes 11% of catalytic residues [3]. Arg occurs more frequently than other basic residues (e.g., lysine) because it has three nitrogen containing groups in the side chain, all of which can perform electrostatic interactions. The side chain of Arg can participate therefore in many electrostatic interactions, and it can be positioned more accurately to facilitate catalysis. The Arg side chain has also a favorable geometry to stabilize a pair of oxygen atoms on a phosphate group (Figure 1), a common biological moiety [3]. Arg in a catalytic center can be involved in various kinds of interactions, for example, electrostatic, hydrogen bond formation, transition state stabilization, activation of water, and the activation of substrates.

If the Arg residue in the active site of the enzymes is replaced by an Arg mimetic, this will cause the loss of enzymatic action, thus disturbing many metabolic pathways. This probably could be the reason for cell disorders. Adenylate kinase (ADK) was chosen as an example in order to explore how Arg analogues will affect enzymatic action. This enzyme catalyzes the reversible reaction

(1) $$\begin{array}{c} 2\;\text{ADP }⇆\text{ATP}+\text{AMP} \end{array}$$

and may process metabolic signals associated with ATP use [23–28]. In this case, ADK has been implicated in the regulation of the metabolically sensitive ion channels and transporters [29–32]. In addition, disruption of the ADK gene impedes the export of ATP from the mitochondria [33]. What will happen if a gene is working but some mutation of ADK appears, such as arginine analogues (Cav, NCav, NCan, NsArg, and sArg) being incorporated instead of Arg in the active site of the enzyme?

In order to study this issue, docking of analogues with arginyl-tRNA synthetase was our choice for the first step. This should show if analogues could be recognized by the enzyme responsible for transportation of arginine residues [34]. Aminoacyl-tRNA constitutes a family of RNA-binding proteins, that is, responsible for the correct translation of the genetic code by covalently linking the appropriate amino acid to the 3′ end of the correct tRNA. In most organisms, there are 20 distinct aminoacyl-tRNAs, with each one of them being responsible for aminoacylating its cognate tRNA with unique amino acid in a two-step catalytic reaction [35]. The mechanism of their action is well known. The synthetase first binds ATP and corresponding amino acid to form acetylamino-AMP. This intermediate interacts with appropriate tRNA molecule, and amino acid is transferred to 3′ end of the tRNA.

## 2. Methods

### 2.1. Enzymes and Arginine Analogues

**Figure 2:**
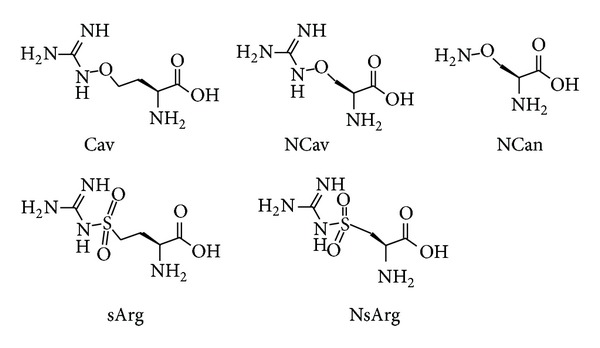
Structures of the investigated arginine analogues.

The protein sequence for human arginyl-tRNAsynthetase (HArgS) was obtained from the UniProt database (accession number Q5T160) [34]. Oxy- and sulfoarginine analogues: l-canavanine (Cav), l-norcanavanine (NCav), l-norcanaline (NCan), l-norsulfoarginine (NsArg) and l-sulfoarginine (sArg), were previously synthesized and biologically tested [36–39] (Figure 2).

### 2.2. Computational Tools

In order to perform computational studies, the following software was used in the present work: compound preparation and homology modeling were done with Molecular Operating Environment (MOE) [40]; docking studies were performed by using Genetic Optimization for Ligand Docking (GOLD 5.1) [41], run on the Scientific LINUX 5.5 operating system [42]; for generation of figures and the exploration of interactions after docking, the Molegro Molecular Viewer (MMV) [43, 44] was applied. 

### 2.3. Homology Modeling

It was based on a single template and was performed with MOE. The best hit was proved with the crystallographic structure of arginyl-tRNA synthetase (ArgS) of *Saccharomyces cerevisiae* (Protein Data Bank [45]-PDB Id: 1f7u) with 40% identity.

### 2.4. Docking of Arginine Analogues

Five oxy- and sulfoanalogues of arginine with already known *in vivo* and *in vitro* biological effects were selected for docking studies. 3D structures of the compounds were modeled with MOE and protonated at physiological pH. Docking was carried out with GOLD 5.1 software. It uses a genetic algorithm and considers full ligand conformational flexibility and partial protein flexibility. The active site of HArgS was defined as residues within 10 Å radius of Glu130, which is responsible for guanidinium group recognition.

### 2.5. Mutations of Adenylate Kinase (ADK)

One arginine residue seems to be very important at the active site of the enzyme—Arg138. Arg175 is very close to the active site, and it could play a role in catalytic ability of the enzyme. Mutations were made in MOE by redrawing the residues and minimizing structures obtained. Docking studies were performed with all mutated enzymes and *bis*(adenosine)-5′-tetraphosphate in order to find out how mutations affect enzymatic action. The structure of adenylate kinase was obtained from RCSB (PDB Id: 2c9y).

## 3. Results and Discussion

### 3.1. Modeling and Docking of HArgS

**Figure 3:**
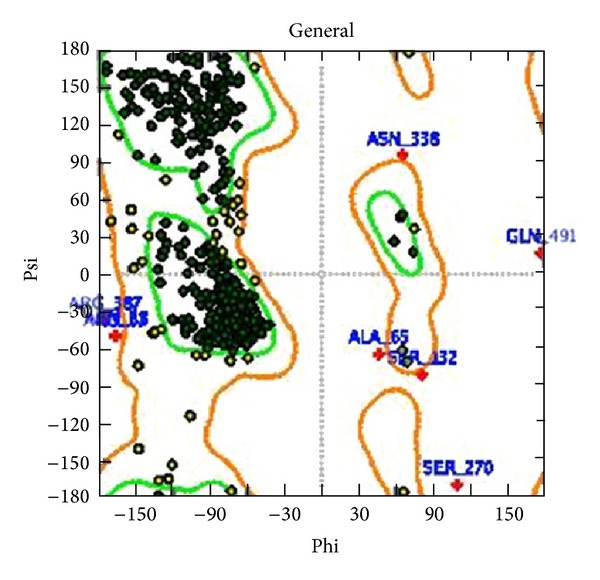
Ramachandran plot of HArgS, obtained from MOE: most favorable regions are −80.8%, allowed regions are −18.3%, and disallowed regions are −0.9%; outliers were presented with +.

Homology modeling of HArgS with a single template was performed by MOE, and a standard molecular mechanics forcefield-amber99 [46] was used. Ten models were generated, and the model was optimized by energy minimization. Phi-psi plot or Ramachandran plot for the chosen model is presented on Figure 3.

Disallowed regions are less than 1%, according to the Ramachandran plot, so it could be used in further studies. The selected model was also checked by docking.

**Figure 4:**
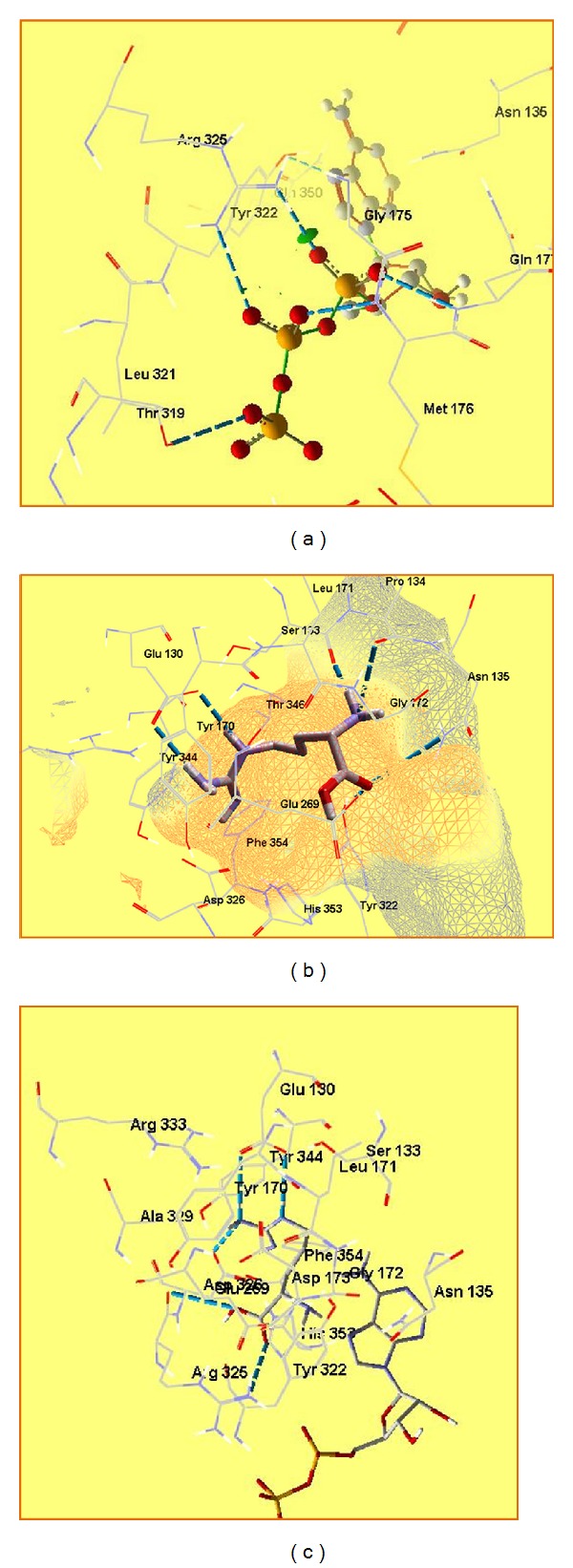
Binding of ATP to HArgS (a), l-arginine to HArgS without ATP (b), and l-arginine to HArgS with ATPc.

It is known [47] that ATP binds to the loop close to the binding site of arginine. This helps the forming of the arginyl-AMP. It interacts electrostatically with Arg325 (Figure 4(a)). When there is no ATP molecule attached to HArgS, arginine binds as shown in Figure 4(b). All the H-bonding capability of the substrate is used by the protein for the specific recognition. The *α*-amino group of arginine forms H-bonds with main chain carbonyls of Ser133 and Phe134, while *α*-carboxylate interacts with the amide nitrogen of Asn135 and phenyl oxygen of Tyr322. Residues Ser133 and Asn135 are very important for correct recognition of *α*-amino and *α*-carboxyl groups. The guanidinium group forms two salt bridges with two carboxylate residues: Glu130 and Asp326. If ATP binds, some conformational changes occur, and *α*-carboxyl group of arginine interacts with Arg325, and this facilitates forming the arginyl-AMP (Figure 4(c)).

Dockings with oxy- and sulfoanalogues of arginine were performed in order to check if they can act as substrates for this enzyme.

**Figure 5:**
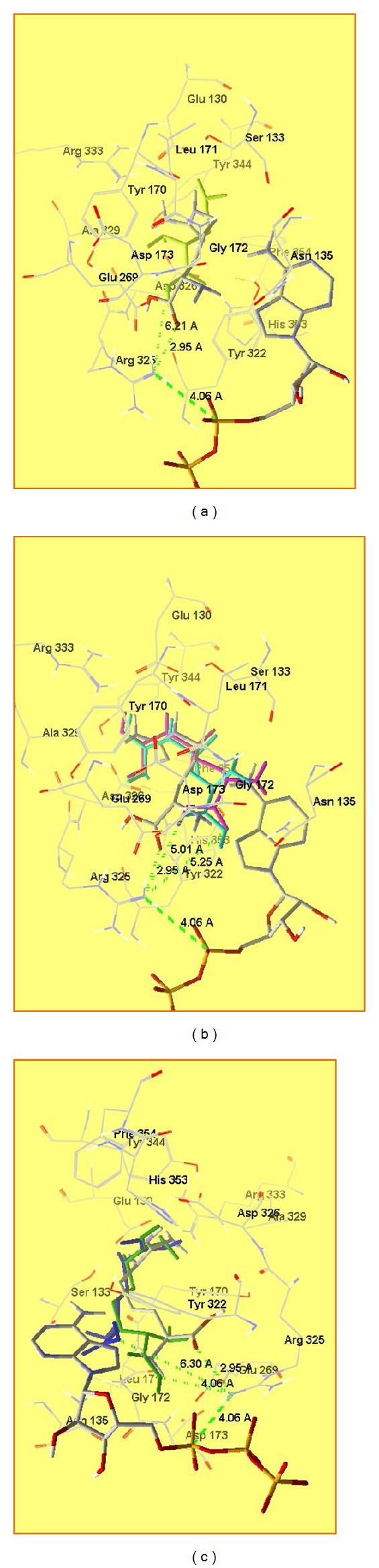
Arginine analogues superposed in the active site: arginine, dark green-Cav, light green-NCan, dark blue-NCav, light blue-NsArg, and purple-sArg.

The interactions between the compounds and enzyme are listed in Table 1. Glu130 is an important site for substrate recognition in the enzyme. It binds to the guanidinium group of arginine. In the molecule of NCan, there is no guanidinium group and such interaction does not appear. Fitness function value for NCan is the lowest in this series of compounds. The distance between its *α*-carboxyl group and Arg326 is too long to interact with it and thus to react successfully with ATP (Figure 5(a) and Table 2).

NsArg has a sulfoguanidinium group, and though it is less polar than arginine's guanidinium group, interaction with Glu130 is still present. In the case of this compound, we have additional interaction of the sulfogroup with Tyr322. The carboxyl group interacts with other residues from the active site, and the distance between this group and Arg325 is too long (Figure 5(b)).

In the case of sArg, the possibility for interaction between sArg and ATP is higher than NsArg as its *α*-carboxyl group is closer to Arg325 (Figure 5(b)).

The enzyme can recognize NCav and Cav as substrates, but only Cav could interact with ATP to form Cav-AMP. The distance between *α*-carboxyl group and Arg325 is too long to have a reaction with ATP (Figure 5(a)).

From this series of arginine analogues, we could mark three of them that could play the role of substrates for HArgS. These compounds could be incorporated in different peptides and protein molecules, and in the present work, we are analyzing the effects of this interaction on adenylate kinase.

### 3.2. Docking of Mutant Adenylate Kinases (ADK)

With the help of MOE, we made mutations at two points subsequently in the active site of ADK-Arg138 and Arg175, with Cav, NCav, and sArg. After energy minimizations of 6 new structures, docking with *bis*(adenosine)-5′-tetraphosphate was performed with GOLD. *Bis*(adenosine)-5′-tetraphosphate could mimic the interactions in the binding sites of ADK as it occupies both sites of the enzyme.

For the recognition of purine moiety of the substrate, very important residue is needed, Arg138, which interacts by a p-*π* interaction.

**Figure 6:**
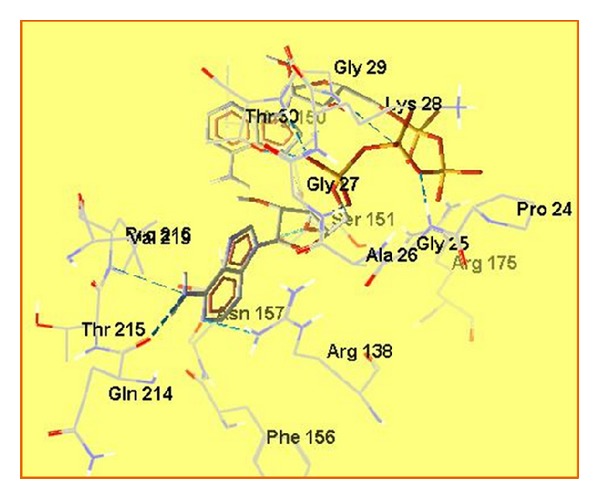
Interactions in the active site of ADK with *bis*(adenosine)-5′-tetraphosphate.

Second important point in enzyme sequence is GAGKG (residues 25–29). It is conserved and fully closed over substrate molecule. It interacts with all phosphate oxygen atoms of ADP molecule by forming hydrogen bonds with backbone amide groups (Figure 6).

**Figure 7:**
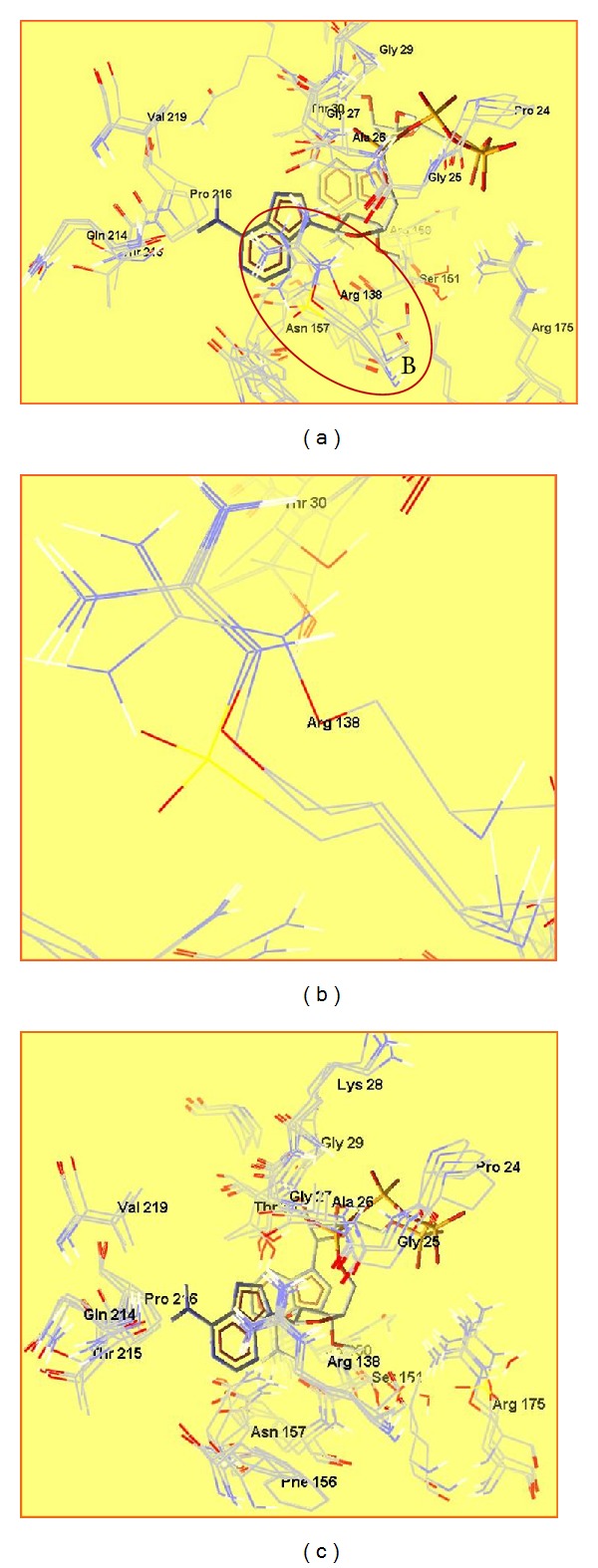
Superposed active sites of ADK and Arg138 mutated ADK with *bis*(adenosine)-5′-tetraphosphate (a); focus on Arg138 residue in Arg138 mutated enzymes (b); superposed active sites of ADK and Arg175 mutated ADK with bis(adenosine)-5′-tetraphosphate (c).

All three analogues of arginine have the guanidinium group as a structural element. In all three cases, it is connected via more electronegative atoms (oxygen and sulfur) than the carbon atom; thus, it is less polar than guanidinium group of Arg. In all three mutations of Arg138 with its analogues, the typical site for ADP recognition still remains, and the purine moiety binds respectively, to oxy-, and sulfoguanidinium group. In the cases of Cav138 and sArg138 mutations, total energies of the enzyme-substrate complexes decrease, while in the case of Cav138 mutation, total energy increases. Fitness function value is almost the same when Arg138 is replaced by Cav, due to their structural similarity, and conformational changes in that case are very small (Figures 7(a) and 7(b)). When Arg138 is replaced by NCav and sArg, conformational changes in the binding site of the enzyme are bigger, and fitness function values are higher which is connected to the binding affinity of the substrate. The higher the fitness function value, the higher the affinity of the substrate to the enzyme and it will bind more strongly. This will decrease the rate of the enzymatic reaction and most probably will stop it in the case of NCan138 and sArg138.

Mutations in position Arg175 do not affect the enzyme much. There are some conformational changes and the values of fitness function indicate that, but newly formed enzyme-substrate complexes have total energies not very different from that of wild type enzyme-substrate complex. These mutations do not affect Arg138 and the binding site for purine moiety remains unchanged (Figure 7(c)).

**Figure 8:**
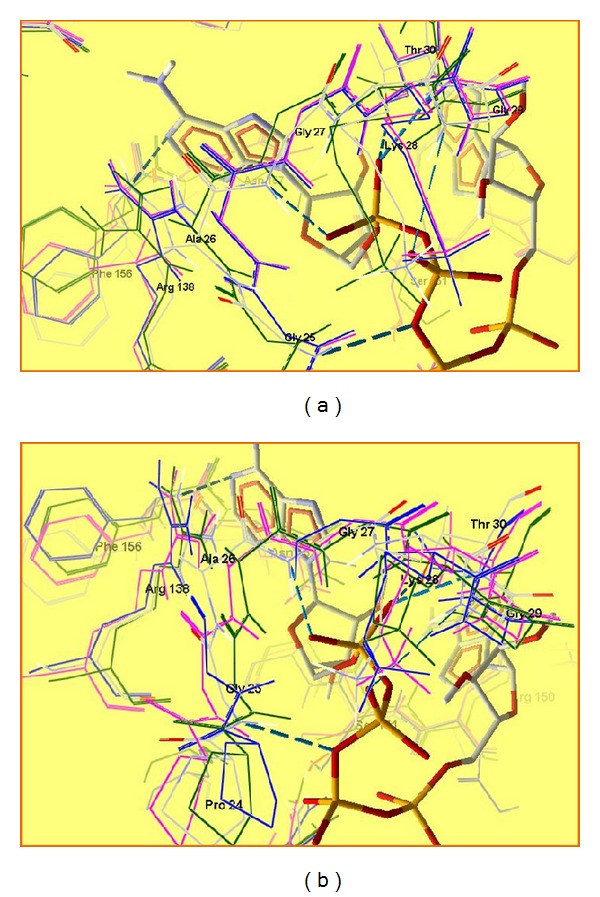
Superposition of the sequence GAGKG (25–29): (a) ADK and ADK mutated with Cav138 (blue), NCav138 (green), and sArg138 (purple); (b) ADK and ADK mutated with Cav175 (blue), NCav175 (green), and sArg175 (purple).

Exploring the sequence GAGKG in the case of Arg138 mutation, the biggest conformational changes occurred when Arg138 is replaced by NCav (Figure 8(a)).

In all mutations in position 175, conformational changes are significant (Figure 8(b)). Purine moiety could be recognized by the mutant enzyme, but because of the changes in the site responsible for interaction between two ADP molecules, reaction could not be performed. These mutations could lead to inactive enzyme.

## 4. Conclusion

From previously synthesized and biologically tested arginine analogues, three of them Cav, NCav, and sArg could act as its antimetabolites. They could be recognized by arginylate-tRNA synthetase as substrates and could be included in numerous biologically important peptides and proteins. Once they become an element of proteins' sequence, for example, enzymes, they would cause crucial changes in their structure leading to loss of enzymatic action. As long as these compounds exhibit a prolonged biological effect, this most probably is due to their incorporation into important metabolic enzymes.

## Figures and Tables

**Table 1:** Fitness function values, interactions with the enzyme, total energies, and energies of binding with the *α*-carboxyl group of the compounds investigated.

Compound	Fitness function	Interactions with the enzyme
Arg	46.4	Two interactions between Glu130-Gu group and Asp326-Gu group and 2 interactions between Arg325-*α*-carboxyl group, Glu269-*α*-carboxyl group, Gly172-*α*-amino group

NCan	32.98	Glu130-oxyamino group, Asp326-*α*-amino group, and His353-*α*-carboxyl group

NCav	44.09	Two interactions between Glu130-oxyGu group, Tyr322, Asn135-*α*-carboxyl group, Tyr170, and Ser133-*α*-amino group

Cav	36.8	Two interactions between Glu130-oxyGu group, Tyr322, Asp135, Gly172-*α*-carboxyl group, and Leu171-*α*-amino group

NsArg	44.04	Glu130, Asp326-sulfoGu group, Asp326, Tyr344-sulfoGu group, Tyr322-SO_2_ group, Ser133-*α*-amino group, Glu269, and Asn135-*α*-carboxyl group

sArg	46.78	Two interactions between Glu130-sulfoGu group, Tyr322-SO_2_ group, Asp135, Gly172-*α*-carboxyl group, and Ser133-*α*-amino group

**Table 2:** Total energies of the complexes and fitness function values of *bis*(adenosine)-5′-tetraphosphate with ADK and mutated ADK.

Mutation	Total energy of the complex	Fitness function value
Without any mutation Arg138 and Arg175	−135.483	69.29
Cav138, Arg175	−112.329	68.08
NCav138, Arg175	−147.651	90.30
sArg138, Arg175	−102.358	97.74
Arg138, Cav175	−128.855	68.08
Arg138, NCav175	−127.472	93.76
Arg138, sArg175	−131.506	96.24
